# Vesicoscopic Ureteral Reimplantation: A Minimally Invasive Technique for the Definitive Repair of Vesicoureteral Reflux

**DOI:** 10.1155/2008/973616

**Published:** 2008-11-05

**Authors:** Venkata Jayanthi, Ashay Patel

**Affiliations:** Section of Urology, Nationwide Children's Hospital, Columbus, OH 43205, USA

## Abstract

The surgical treatment of vesicoureteral relfux can range from injection therapy to open ureteral reimplantation. Minimally invasive applications for treatment of vesicoureteral relfux include laparoscopic extravesical and intravesical ureteral reimplantation. We present our extended experience of the technique for intravesical cross-trigonal ureteral reimplantation for vesicoureteral relux.

## 1. INTRODUCTION

As in all areas of surgery, there is an ever increasing interest in minimally invasive techniques. Injection therapy using dextranomer/hyaluronic acid is a simple technique with low morbidity but most studies would suggest that this approach is not as successful as standard repair. Laparoscopic reconstructive surgery, for whatever underlying pathologic condition, has the expectation and advantage that as one tries to follow the same principles as with open repair, after the learning curve period, success rates should be identical.

Most reports of laparoscopic repair of reflux have described the use of an extravesical technique with relatively good success rates. Many urologists however prefer to correct reflux using an open transvesical approach. The feasibility to replicate this technique using a vesicoscopic approach was demonstrated by Gill et al. [1] Yeung however was the first to present a large series of patients undergoing cross-trigonal ureteral reimplantation using CO_2_ pneumovesicum with success rates nearly identical to standard open repair [2]. Simiarly, Valla et al. reported their experience with this technique again demonstrating high success rates [3]. Kutikov et al. presented their initial experience with vesicoscopic reimplantation for both primary reflux and megaureter repair [4]. A retrospective review from our center has demonstrated decreased pain in patients undergoing a vesicoscopic approach compared to standard Cohen repair [5]. In this report, we present our extended experience with vesicoscopic cross-trigonal ureteral reimplantation.

## 2. MATERIALS AND METHODS

### 2.1. Patient selection

Our preference is to use this technique only in children with primary reflux (less than grade IV) who have seemingly normal bladder function based on clinical history or have dysfunctional elimination syndrome responsive to standard treatments. Though there are some published reports of using a vesicoscopic technique for megaureter repair, we have elected to use this technique only in situations where tapering would not be needed. We have performed this procedure in children as young as 13 months, but there realistically may not be much of an advantage in performing vesicoscopic repair in children less than 2 years of age. The decreased working space in younger children does make the procedure more technically demanding and may obviate the advantages of vesicoscopic repair. Preoperative bladder volume was not utilized to evaluate inclusion criteria for surgical consideration. Failed injection therapy does make dissection more complicated but should not be considered a contraindication.

### 2.2. Surgical technique

**Positioning**

**Figure 1:**
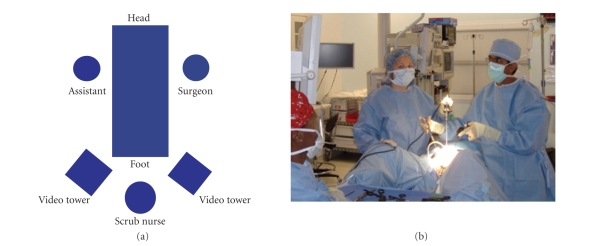
Patient is placed in dorsal lithotomy position with the surgeon standing to the patient's left looking at a monitor over the right leg.

The procedure is performed with the child in the dorsal lithotomy position with the abdomen and perineum within the sterile field (Figure 1). Urethral access is needed at various times during the procedure. Due to the extended length of the procedure, careful positioning and padding of the legs is needed to prevent nerve palsy.

The surgeon typically stands on the patient's left side with the monitor positioned over the right leg. The assistant, that is, camera holder, stands on the patient's right looking at a monitor positioned over the left leg. The scrub nurse typically stands between the legs.

**Bladder wall fixation and port placement**

**Figure 2:**
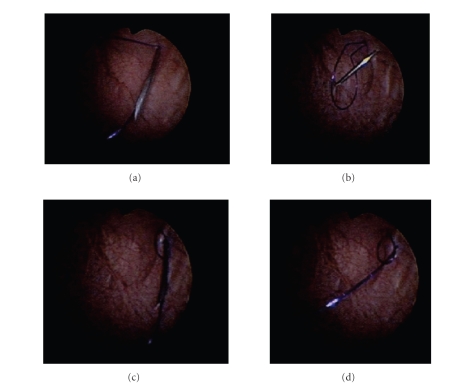
(a) Spinal needle has pushed suture into bladder creating pulling loop. (b) Spinal needle passed through pulling loop via an adjacent puncture. (c) With needle through pulling loop, one free end of the suture is passed through spinal needle and thus the pulling loop. (d) Removal of spinal needle results in suture being snared by pulling loop. Subsequent retraction of pulling loop creates through-and-through suture which can then be tied fixing bladder to abdominal wall.

After positioning the patient, using a pediatric cystocope rigid cystourethroscopy is performed using a 30-degree lens during the fixation of the bladder wall. Fixation of the bladder to the anterior abdominal wall is critical for several reasons. Firstly, it can be difficult to push a port through fascia and bladder wall. Fixation of the bladder will create enough resistance to allow ports to be more easily introduced. Secondly, in case of inadvertent removal of the port during the procedure, having the bladder fixed to the abdominal wall will maintain the relationship between the skin incision and the entry site within the bladder permitting replacement of the port. Pneumovesicum is created using CO_2_ introduced through the irrigation port of the cystoscope at maximal pressures of 10–15 mm Hg. Once the bladder is maximally distended, under cystoscopic guidance the dome and lateral walls of the bladder are fixed to the abdominal wall. The present technique for placement of the fixation sutures is adapted from a report on percutaneous internal ring suturing, a method for percutaneously closing the patent processus vaginalis in children with inguinal hernias or communicating hydroceles [6]. Briefly, a 2-0 PDS suture is placed through an 18 guage spinal needle. Under cystoscopic guidance, the spinal needle is introduced into the bladder (Figure 2(a)). This will naturally push the suture into the bladder. Upon extraction of the needle, a loop of suture, called the pulling loop, will be left in the bladder. Through an adjacent puncture, the spinal needle is inserted into the bladder and through the pulling loop (Figure 2(b)). One end of the suture that formed the pulling loop is then inserted through the needle, thus placing it through the loop (Figure 2(c)). Retracting the pulling loop out of the bladder pulls the free end of the suture creating a through-and-through suture which can be tied fixing the bladder to the abdominal wall (Figure 2(d)). Fixation sutures are placed in the midline as well as the lateral walls of the bladder. A 5 mm port is placed in the midline for the camera and two 3 mm ports placed laterally for the working ports. These ports are placed immediately distal to the fixation sutures in the direction of the bladder neck. It is often helpful to place a purse string suture around the ports to further immobilize them, minimizing the chances for inadvertent removal. For most children, 3 mm laparoscopic instruments that are 20 cm in length are ideal.

**Ureteral dissection**

**Figure 3:**
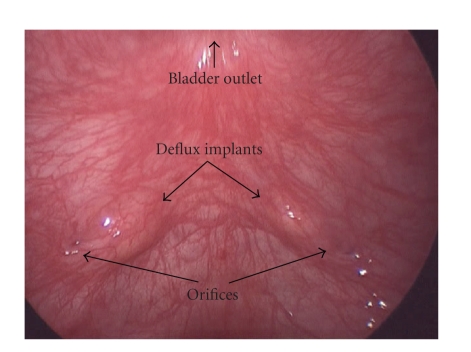
Initial “vesicoscopic” view of operative site in a patient that had failed prior injection therapy.

**Figure 4:**
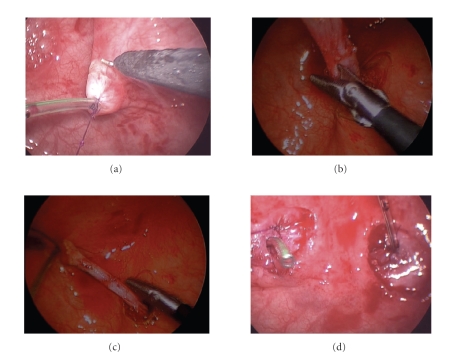
(a) Initial dissection with hook electrode at low-power setting. (b) Investing detrusor bands divided using sharp dissection. (c) Ureter has been mobilized such that it can reach the contralateral side with no tension. (d) View after bilateral mobilization and closure of the posterior detrusor openings. Ureters have been pushed back out of bladder to permit visualization of the bladder mucosa-detrusor plane to permit creation of the submucosal tunnels.

Vesicoscopy is performed using a 5 mm 30-degree lens. The orientation is such that the bladder neck will be located at the 12:00 position (Figure 3). Feeding tubes (3.5 Fr.) are placed per urethra, passed up each ureter, and fixed with fine suture. Dissection is begun by using a hook electrode at a power setting of 10 (low power) (Figure 4(a)). Lifting up on the suture holding the feeding tube in place will create sufficient tension such that incision of the bladder mucosa with the hook electrode will cause the bladder to fall back. In a manner analogous to open transvesical surgery, the ureter can be mobilized from the surrounding detrusor muscle using a combination of sharp and blunt dissection. Extreme care must be used when transecting investing bands of detrusor and it may be safer to divide these bands sharply as opposed to using cautery (Figure 4(b)). This dissection is rather easy and rapid in children with thin-walled bladders but can be quite difficult if a child has a markedly thickened bladder wall. Dissection is continued until enough length is gained to bring the ureter to the contralateral side (Figure 4(c)). The posterior detrusor opening is then closed with interrupted 4-0 polydioxanone suture. For bilateral repairs, the contralateral ureter may then be mobilized (Figure 4(d)).

During the procedure a suction device is needed to remove not only blood but also urine that may accumulate at the bladder base. Some authors have left a small urethral catheter indwelling to assist with suction but our preference is to simply use a 3 mm suction-irrigation device through one of the working ports as needed.

**Tunnel creation**

**Figure 5:**
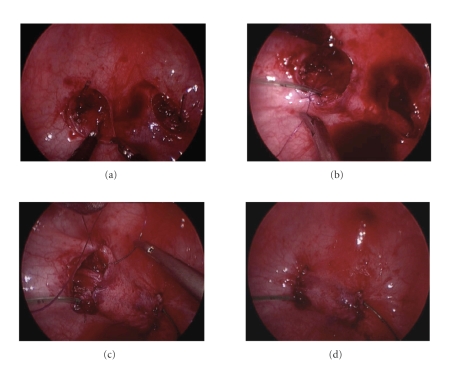
(a) Creation of the submucosal tunnels started by gently lifting up on mucosa and sharp dissection of the appropriate plane. (b) The right ureter has been passed through the tunnel and sutured to the original hiatus on the contralateral side. (c) Both ureters have been transposed and sutured in place. The left mucosal opening is then closed with absorbable suture. (d) Completed repair prior to removing feeding tubes.

Cross-trigonal tunneling is then performed with a combination of blunt and sharp dissection in the submucosal plane (Figure 5(a)). Maryland graspers are used to elevate the mucosa and fine scissors are used to initiate and develop the plane. The positive pressure within the bladder along with the optics of the 30-degree lens can assist with the visualizing the appropriate plane. The length of the tunnel created spans from the initial hiatus across to the contralateral hiatus. After creation of the tunnel(s), the ureters may be placed in the tunnels and passed to the other side. The ureter(s) is then fixed in place with 5-0 polydioxanone suture (Figure 5(b)). The remaining mucosal openings are then closed with absorbable sutures and the feeding tubes removed (Figures 5(c) and 5(d)).

**Bladder port closure**

To maintain the pathway through the incision into the bladder, a feeding tube is placed through each port prior to its removal. Under cystoscopic guidance, the bladder ports are closed using sutures placed in a manner analogous to the initial fixation sutures. After placing the bladder port closure sutures, a foley catheter is inserted to decompress the bladder and the fixation sutures are removed. This allows the bladder to fall away from the abdominal wall. The bladder port sutures are then carefully tied and the skin incisions subsequently closed.

The foley catheter is typically removed in 36 hours. Followup imaging included renal ultrasonography at one month and cystography at 3 months.

## 3. RESULTS

To date, 103 children have undergone attempted vesicoscopic correction. Due to poor port placement, three were converted to open repair leaving a total of 100 patients who did undergo vesicoscopic ureteral reimplantation. There were 91 girls and 12 boys with ages ranging from 13 months to 18 years. Grade of reflux ranged from I to IV. Ten of these children had failed injection therapy with dextranomer/hyaluronic acid. Seventy-eight underwent bilateral repairs and 25 unilateral. Twelve of these patients had duplex systems and underwent common sheath reimplants.

To date, 77 patients have undergone postoperative cystograms and 72/77 (94%) had normal studies. One of these with persistent reflux developed contralateral reflux after unilateral reimplantation. The other four occurred early in the series, within the first 30 patients. Cystoscopy in three of these demonstrated either small ureterovesical fistulae or an absent intramural ureter, suggestive of ischemic injury. Subsequent modification of the ureteral dissection technique has led to no further cases of persistent reflux in the last 47 post-operative cystograms performed.

Two patients did develop postoperative ureteral obstruction requiring temporary percutaneous nephrostomy tube placement. These patients had imaging studies that suggested extrinsic compression from retrovesical urinomas. One patient underwent reoperative ureteral reimplantation at another center and one resolved with stent placement. One patient developed small bladder stones which passed spontaneously. The first patient in the series, who did not have the bladder ports closed separately, did develop a small extraperitoneal leak which healed with bladder drainage. All subsequent cases have had bladder ports closed with no further port site leaks.

Intraoperative complications included proximal ureteral migration of the feeding tubes in four patients necessitating immediate ureteroscopy for retrieval. Pneumoperitoneum occurred occasionally and was treated by intraoperative intraumbilical Veress needle placement.

## 4. DISCUSSION

There is an ever increasing interest in the application of minimally invasive techniques for surgical reconstruction. In many centers there is a wealth of experience in the laparoscopic management of such diverse conditions such as impalpable testes, nonfunctional kidneys, ureteropelvic junction obstruction, and duplex anomalies. However, very few centers have attempted laparoscopic correction of vesicoureteral reflux. There are many possible reasons for this. First and foremost is that standard open surgical correction works so well. It has an extremely high success rate with minimal morbidity. Furthermore, cosmesis is not an issue as typically a small transverse suprapubic incision is required.

If standard ureteral reimplantation is so effective with such minor morbidity, why consider laparoscopic, or rather a vesicoscopic approach? We feel that there may be several advantages. Firstly, we have shown in a retrospective report that patients undergoing vesicoscopic repair have decreased analgesic requirements compared to after open repair. Secondly, it has been our observation that parents are often much more accepting of having definitive surgical correction for their children if they know it will be done “laparoscopically.” Thirdly, in a training center, vesicoscopic reimplantation can be very effective at developing and teaching high-level surgical techniques since careful dissection and fine suturing need to be done, and all within the confines of the bladder.

The ultimate benefit of a surgical procedure must be decided based on a review of the surgical success and rate of complication. After utilizing a very similar technique, Yeung et al. demonstrated results equivalent to open ureteral reimplantation (96% VUR resolution) in a smaller series in children. Valla et al. demonstrated success rates of 92%. Kutikov et al., detailing their early experience, had a 93% success rate. Our present overall success rate is at 94%. However, all of our failures occurred in the first half of our series. Cystoscopic evaluation of the failures demonstrated evidence of possible ischemic injury to the ureters. We subsequently modified our dissection technique and have had no further failures in the last 47 patients tested. Thus with experience gained and lessons learned, we think that vesicoscopic reimplantation is essentially equivalent to open Cohen reimplantation with regard to efficacy of correcting reflux.

Ureteral obstruction may be the most feared complication with ureteral reimplantation and, at least with open surgery, is usually due to ischemic stricture formation or inappropriate angulation through the detrusor neohiatus. In our series we did have two patients with postoperative obstruction related to retrovesical urinomas. We suspect this was due to improperly performed ureterovesical anastamoses with leakage of urine through submucosal tunnel.

Though there are some reports on the use of a vesicoscopic approach for megaureter repair, we have elected not to do this. Firstly, in our experience, it is very rare to need to taper a ureter in the first place. Secondly, a carefully performed tapered reimplantation is difficult enough and in a training institution, our preference is to ensure that our residents and fellows can do a quality open megaureter repair.

With the experience gained in this series, we have applied certain modifications to improve the procedure and its outcomes. Great care during the dissection and mobilization of the affected ureters is necessary to prevent ureteral injury. A low power setting on the hook electrode is mandatory. As there is no fourth port for an assistant, one has to be careful when using electrocautery that the tissue being divided is well away from the ureter.

Port placement can be tricky. If placed too inferiorly, the ports will be right on the orifices. If placed too cephalad, the ports may traverse the peritoneum. Leakage of gas into the peritoneal cavity can occur and the subsequent pneumoperitoneum can lead to collapse of the bladder and poor visibility. Transumbilical Veress needle placement will vent the carbon dioxide and allow the bladder to distend appropriately.

Extraperitoneal urinary leakage diagnosed after the first procedure leads to the inclusion of bladder port closing sutures as outlined earlier. Since the application of this technique, no other port leaks were observed. Migration of the feeding tubes proximal to the ureteral orifice was a problem encountered four times in the study. Occasionally, the suture can pull through the ureteral orifice with traction during dissection or manipulation of the ureter. Fixation of the feeding tube to the ureteral orifice is mandatory to prevent migration of the tube. Occasionally, this requires stopping the dissection to resuture the feeding tube to the distal ureter.

Vesicoscopic ureteral reimplantation is an admittedly challenging procedure. There is a tremendous learning curve and one must exercise a great deal of dedication at wanting to learn the procedure. Though the complication rate that we note in our series is greater than that which may be seen in a contemporary series of open repairs, we suspect that this is an indication of the difficulty in learning the procedure. The adverse events that we have noted in our series are probably due to suboptimal execution of the technique rather than the concept of vesicoscopic reimplantation itself. Our positive experience in the last half of the series is indicative of the fact that vesicoscopic ureteral reimplantation is a highly effective, minimally invasive approach for the definitive repair of primary reflux.
